# Photosynthetic activity in the heterotrophic plant genus *Cuscuta* (Convolvulaceae) is modulated by phylogeny and ontogeny

**DOI:** 10.1093/aob/mcaf145

**Published:** 2025-07-09

**Authors:** Adam C Schneider, Jenna T B Ekwealor, Ariana Besik, Nurulain Ibrahim, Ingo Ensminger, Saša Stefanović

**Affiliations:** Department of Biology, University of Toronto Mississauga, Mississauga, ON L5L 1C6, Canada; Department of Biology, University of Wisconsin–La Crosse, La Crosse, WI 54601, USA; Department of Biology, San Francisco State University, San Francisco, CA 94132, USA; Data Science Lab, Office of the Chief Information Officer, Smithsonian Institution, Washington, DC 20002, USA; Department of Biology, University of Toronto Mississauga, Mississauga, ON L5L 1C6, Canada; Department of Cell and Systems Biology, University of Toronto, Toronto, ON M5S 1A1, Canada; Department of Biology, University of Toronto Mississauga, Mississauga, ON L5L 1C6, Canada; Department of Biology, University of Toronto Mississauga, Mississauga, ON L5L 1C6, Canada; Department of Cell and Systems Biology, University of Toronto, Toronto, ON M5S 1A1, Canada; Department of Ecology and Evolutionary Biology, University of Toronto, Toronto, ON M5S 3B2, Canada; Department of Biology, University of Toronto Mississauga, Mississauga, ON L5L 1C6, Canada; Department of Ecology and Evolutionary Biology, University of Toronto, Toronto, ON M5S 3B2, Canada

**Keywords:** Carotenoids, chlorophyll, chlorophyll fluorescence, comparative phylogenetics, *Cuscuta*, lutein epoxide cycle, parasitic plants, photosynthetic activity, xanthophyll cycle

## Abstract

**Background and Aims:**

Photosynthesis is central to plant function, yet it has been repeatedly lost or diminished in parasitic angiosperm lineages. This variation raises questions about how photosynthetic function is retained, modified or repurposed in the evolutionary context of parasitism. *Cuscuta* species, as a model system for studying parasitism, exhibit varying degrees of plastid functionality and photosynthetic ability, based on genomic and ultrastructure studies. However, few direct physiological studies exist, and none that span multiple developmental stages of autotrophic, mixotrophic and non-photosynthetic species in a phylogenetic framework.

**Methods:**

To address this gap, we paired photosynthetic activity measurements from fluorometry with quantitative analysis of chlorophylls and carotenoids from multiple developmental stages in 14 *Cuscuta* species, representing the phylogenetic breadth of the genus, and a closely related autotrophic species. Multivariate data were analysed using non-parametric hypothesis tests, and comparative phylogenetic patterns were explored through Bayesian model testing.

**Key Results:**

Photosynthetic activity and chlorophyll and carotenoid content were highest in meristematic regions (e.g. shoot tips and developing seeds) and lowest in older stems or haustoria. Neoxanthin, a carotenoid typically highly conserved in plants, appears to have been lost once in *Cuscuta* and subsequently re-gained in certain lineages. Complex relationships between photosynthetic activity and lutein epoxide concentration suggest differing roles in developmental stages with high and low energetic needs.

**Conclusions:**

These findings provide substantial evidence that photosynthesis in *Cuscuta* is not vestigial but rather modulated based on developmental stage and across phylogenetic history, revealing a dynamic interplay between parasitism and photosynthetic function.

## INTRODUCTION

While photosynthesis is a defining feature of plant life, 1–2 % of angiosperms have completely or partially abandoned this vital process, turning to parasitism of carbon, energy and nutrients from other organisms. This shift, observed across multiple lineages, is associated with drastic changes in vegetative morphology (Kuijt, 1969; Leake, 1994; Joel *et al*., 2013; Balios *et al*., 2025), genomic architecture (Graham *et al*., 2017; Sun *et al*., 2018; Vogel *et al*., 2018; Yoshida *et al*., 2019; Lyko and Wicke, 2021; Chen *et al*., 2023) and photosynthetic physiology (Stewart and Press, 1990; Těšitel, 2016).

The degree of host dependence and the reduction of photosynthetic capacity in parasitic plants vary significantly both within and among the several dozen independently evolved lineages (Merckx, 2013; Nickrent, 2020). In flowering plants, heterotrophy ranges from facultative mycoheterotrophy such as in some orchids (Cameron *et al*., 2009) to obligate holoparasitism in families such as Balanophoraceae and Rafflesiaceae (Su *et al*., 2015; Barkman *et al*., 2017). These transitions often involve a reduction or complete loss of photosynthetic function, but often some vestigial traits associated with photosynthesis remain (e.g. plastid-encoded *rbcL* transcription; van der Kooij *et al*., 2000).

**Fig. 1.**
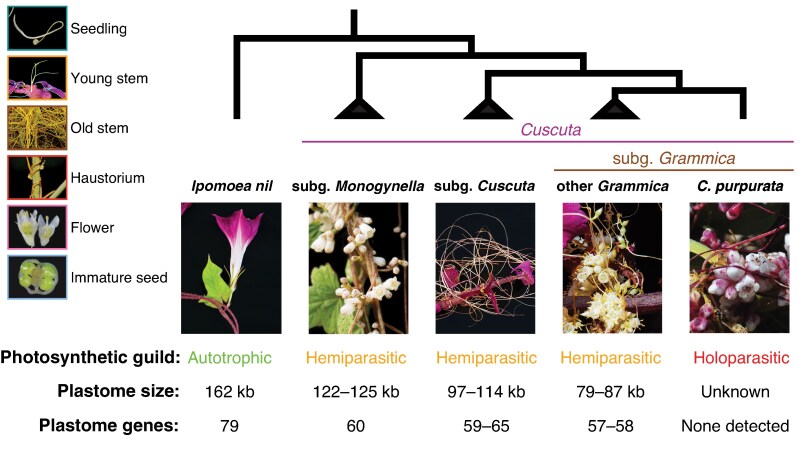
Representative photos of the six tissue types/ontogenetic stages sampled in this study, and the phylogenetic relationships, reliance on photosynthesis and plastome differences among major clades of *Cuscuta* and close relative *Ipomoea nil.* Plastome references: Hoshino *et al*. (2016), Funk *et al*. (2007), McNeal *et al*. (2007), Banerjee and Stefanović (2020, 2023). Photo credits are given in the Acknowledgements.

*Cuscuta* L. species (dodders; Convolvulaceae; Fig. 1; Supplementary Data Fig. S1) in particular illustrate the complex nature of photosynthetic loss, which is not always straightforward and cannot be fully explained by a reductionistic focus on single genes or proteins. For instance, no detectable levels of rubisco expression were found in an undetermined *Cuscuta* subgen. *Grammica* species (misidentified as *C. europaea*), despite the presence of the rubisco-encoding *rbcL* gene (Machado and Zetsche, 1990). In some cases, *Cuscuta* species lack plastid-encoded polymerase (*rpo*) genes but retain plastid *rbcL* transcription due to nuclear-encoded promoters (Krause *et al*., 2003; Berg *et al*., 2004). Conversely, *C. reflexa* is capable of low levels of photosynthetic carbon assimilation in its noticeably chlorophyllous stems despite loss of all *ndh* genes (Haberhausen and Zetsche, 1994). It is hypothesized that this species photosynthesizes only to recycle carbon dioxide generated from metabolizing carbohydrates originating in its host plant, suggested by the restriction of chlorophyll production to a specific layer of cells surrounding the stem vascular bundles where it is isolated from atmospheric gas exchange (Hibberd *et al*., 1998). Finally, other *Cuscuta* species have chlorophyll *a*:*b* ratios comparable to autotrophic plants along with properly localized photosynthetic proteins yet still maintain only low levels of carbon assimilation (Sherman *et al*., 1999; van der Kooij *et al*., 2000). Such studies have provided valuable insight into photosynthetic physiology and molecular evolution of *Cuscuta*; however, they were limited to one or a few species and generally did not explore developmental context. That is, functional studies of photosynthetic physiology have focused primarily on mature stems, where parasitism is well established and endogenous carbon fixation plays a less critical role. Other life stages may require different energetic needs, such as during seedling establishment, host attachment or reproduction. Field and laboratory observations on select hemiparasitic *Cuscuta* species have indicated variation in the intensity of green tissue, particularly at shoot tips, haustoria and inflorescences (maturing ovules), and that coloration varies with host or environmental conditions (Lyshede, 1985; Qin *et al*., 2008). Indeed, one of the most striking features of many species of *Cuscuta* is pale to intense yellow or orange coloration due to chlorophyll reduction and intense carotenoids (Yuncker, 1932; van der Kooij *et al*., 2000). Investigating how photosynthesis varies across organs and developmental stages could provide insight into why many *Cuscuta* species, despite being fully dependent on host plants for survival, retain some photosynthetic capacity.

While carotenoids generally perform the same photoprotection and photoregulation roles in *Cuscuta* as in other plants (Choudhury and Sahu, 1999; Snyder *et al*., 2005), several species in *Cuscuta* have a unique xanthophyll cycle that contributes to photoprotection and photoregulation by dissipating excess light energy. Known as non-photochemical quenching (NPQ), this set of processes is otherwise highly conserved in plants (Baccarini *et al*., 1965; Hibberd *et al*., 1998; Bungard *et al*., 1999; van der Kooij *et al*., 2000; Qin *et al*., 2008). Specifically, in *C. reflexa* and several congeners, 9-*cis* violaxanthin has replaced neoxanthin (presumably 9-*cis* neoxanthin) in the light-harvesting complex (Takaichi and Mirauro, 1998; Snyder *et al*., 2004), with implications that are not fully understood. This violaxanthin-based process happens in addition to the lutein epoxide cycle, which is a second xanthophyll cycle that contributes to NPQ and possibly increases the light-harvesting efficiency of photosystem II (PSII), especially in high-light environments, both of which could aid in the adaptation of *Cuscuta* to diverse ecological niches or offset the loss of several key nuclear genes that support photosynthesis at high light intensities (Snyder *et al*., 2005; García Plazaola *et al*., 2007; Kruk and Szymanska, 2008; Vogel *et al*., 2018). However, no comprehensive comparisons have been made between carotenoid composition and photosynthetic activity across species and clades within *Cuscuta*. Furthermore, understanding the extent of neoxanthin loss and modulation of the lutein epoxide cycle across the phylogeny and at different developmental stages within *Cuscuta* may shed light on the relative timing and adaptive significance of these evolutionary changes relative to photosynthetic shifts concomitant with parasitism.

Despite progress in understanding the evolution of parasitism, gaps remain in linking photosynthesis and pigment loss to the evolutionary trajectories of parasitic plants. To address these gaps, this study examined how *Cuscuta* species balance photosynthetic function and parasitism by quantifying pigment composition and photosynthetic activity across phylogenetic and developmental contexts. By integrating phylogenetics with ontogenetic analysis, we sought to uncover the evolutionary mechanisms governing photosynthetic retention and loss in parasitic plants. Our study, comprehensive both taxonomically and ontogenetically, investigated pigment composition and photosynthetic activity in the model clade *Cuscuta*, with three specific purposes in mind: (1) to characterize how phylogeny and ontogeny affect the function of the light reactions and pigment composition, (2) to infer the relative timing, extent, and reversibility of neoxanthin loss in *Cuscuta*, and (3) to understand the relationship between lutein epoxide abundance and photosynthesis activity in different tissues of *Cuscuta*.

## MATERIALS AND METHODS

### Study system: *Cuscuta* as a model clade

The phylogenetic and functional diversity within *Cuscuta* provides an excellent system to investigate the evolutionary dynamics of photosynthetic retention and loss in parasitic plants. Thus, *Cuscuta* serves as a model clade (*sensu*  Howarth and Dunn, 2016) for understanding trophic shifts in plants, due to the convergence of three main factors (Fig. 1). First, phylogenetic relationships within this species-rich genus are well-resolved and strongly supported. This includes backbone support for four monophyletic subgenera *Monogynella*, *Cuscuta*, *Pachystigma* and *Grammica*, and resolution nearer the tips, with ca. 200 species circumscribed into 19 monophyletic sections (Garcia *et al*., 2014; Costea *et al*., 2015). Second, the genus *Cuscuta* is one of only two clades of haustorial angiosperms known to span the trophic continuum from photosynthetic hemiparasites to non-photosynthetic, obligate holoparasites (Nickrent, 2020). Most of its constitutive species have been called ‘cryptically photosynthetic’ (McNeal *et al*., 2007), meaning they are capable of limited and localized photosynthesis (Hibberd *et al*., 1998; van der Kooij *et al*., 2000). However, some species from sect. *Subulatae* are achlorophyllous, lack thylakoids and do not produce detectable levels of rubisco protein or the transcript of its large subunit, *rbcL* (van der Kooij *et al*., 2000). These species, as well as those of another separate putatively non-photosynthetic clade, sect. *Ceratophorae* pro parte, show substantial deletions in plastid gene content (Braukmann *et al*., 2013; Banerjee and Stefanović, 2019, 2020 , 2023). Third, the shallow phylogenetic (i.e. infrageneric) level that encapsulates this trophic diversity minimizes potentially confounding historical factors, which often emerge when comparing taxa at deeper phylogenetic levels, thereby making this system more tractable.

### Experimental design

Fourteen species of the obligate stem parasite *Cuscuta* and one outgroup species were grown from seed under common conditions (Table 1), evenly spanning the phylogeny of the genus (Supplementary Data Fig. S1) and including the putatively non-photosynthetic *C. purpurata*. While the closest photosynthetic sister group to *Cucuta* remains unresolved (Stefanović and Olmstead, 2004), our outgroup was the closely related autotrophic species *Ipomoea nil* (L.) Roth (morning glory; Convolvulaceae).

**Table 1.** *Cuscuta* and outgroup taxa and vouchers used for fluorescence imaging and pigment analysis. Classification *sensu*  García *et al.* (2014). The number of replicate measurements varies across developmental stage, but individual species–tissue combinations with low replication are only included in the by-clade analyses. An asterisk indicates specimens grown from seed at the University of Toronto Mississauga glasshouse and pressed into a voucher. Herbarium abbreviations according to Index Herbariorum. GenBank accessions indicate the sequences used to build the phylogeny for comparative phylogenetic analyses and did not come from the same individuals as those in our physiological studies. A dash (–) in the last column denotes no sequence data available.

Genus/subgenus	Section	Species	Replicate measurements		Voucher; provenance (Herbarium)	GenBank accessions (*rbcL*, 26S)
			PAM	Pigments		
*C.* subg. *Cuscuta*	sect. *Cuscuta*	*C. epithymum* (L.) L.	5–13	4–7	*M. Costea s.n.*, 11 Mar. 2018; Spain (WLU)	KJ436643, KJ400081
*C.* subg. *Grammica*	sect. *Californicae*	*C. californica* Yunck.	4–42	6–7	*Stefanović SS-17-105;* California, USA (TRTE)	EU883446, EU883494
	sect. *Ceratophorae*	*C. costaricensis* Yunck.	12–14 (1 for seedling)	5–8	*M. Costea s.n.*, 7 Feb. 2018; Michoácan, Mexico (WLU)	KJ436634, KJ400071
	sect. *Cleistogrammica*	*C. australis* Hook.f.	6–10 (2 for seed)	2–3 (1 for seed)	Commercial seed source; UTM-1382* (TRTE)	KJ436607, KJ400044
		*C. polygonorum* Engelm.	4–14	2–5	*Lutz s.n.*, 13 Aug. 2016; Iowa, USA (WLU)	*C. obtusiflora*: KJ436693, KJ400140
		*C. sandwichiana* Choisy^a^	6–14	2–5	*Degener & Degener 36596*; Hawaii, USA (WTU)	–, KJ400165
	sect. *Denticulatae*	*C. denticulata* Engelm.	15–26	2–7 (1 for seed)	*Stefanović SS-13-46*; California, USA (TRTE)	KJ436639, KJ400077
	sect. *Gracillimae*	*C. gracillima* Engelm.	8–17	2 (1 for haustorium)	*M. Costea s.n.*, 6 Feb. 2018; Mexico, Mexico (WLU)	KJ436653, KJ400096
	sect. *Indecorae*	*C. indecora* Choisy	4–18	2–4	*Stefanović SS-16-77*; New Mexico, USA (TRTE)	KJ436665, KJ400109
	sect. *Lobostigmae*	*C. tasmanica* Engelm.	21–36 (3 for seedling)	5–11	*Stefanović SS-17-15*; Tasmania, Australia (CANB, TRTE)	*C. tinctoria:* KJ436721, KJ400175
	sect. *Oxycarpae*	*C. cephalanthi* Engelm.	6–41	4–14 (2 for flowers)	UTM-1567*; Iowa, USA (TRTE)	KJ436618, KJ400054
		*C. compacta* Juss.	4–8	2–6 (1 for flowers)	UTM-1566*; Texas, USA (TRTE)	KJ436626, KJ400064
	sect. *Subulatae*	*C. purpurata* Phil.	4–18	3–6	*Muñoz 5132*; Chile (WLU)	–, KJ400159
*Cuscuta* subg. *Monogynella*	sect. *Monogynella*	*C. lupuliformis* Krock.	4 (seedling only)	4 (seedling only)	*Á. Lovas-Kiss s.n.*, 10 Sep. 2018; Hungary (TRTE, WLU)	KJ436680, KJ400126
		*C. monogyna* Vahl.	4–14	5–9 (2 for flowers)	UTM-1348*; Israel (TRTE)	KJ436686, KJ400133
*Ipomoea*		*I. nil* Phil.	6–13	5–8	Commercial seed source; no voucher	MG973745, *I. lacunosa*: AF146016

^a^ *C. sandwichiana* is probably an intersectional hybrid (Garcia *et al*., 2014). Classification to sect. *Cleistogrammica* is based on the plastid genome, while its nuclear genome places it with sect. *Grammica*.

*Cuscuta* seeds were collected from wild populations (for the provenance of material and voucher information, see Table 1), while *Ipomoea nil* was grown from commercially available seed. *Cuscuta s*eeds were scarified in concentrated (18.4 M) sulphuric acid for 1 min, rinsed at least four times with deionized water, plated on damp filter paper in a culture dish and incubated at room temperature until germination. Individual *Cuscuta* seedlings were transferred to separate mature coleus (*Coleus scutellarioides* L. Benth.; Lamiaceae) host plants. Each *Cuscuta* + coleus pair was grown in a separate pot and considered a biological replicate. In total, we grew three to five individuals per *Cuscuta* species, except for *C. gracillima* (*n* = 2). Because of difficulty in replication, we included *C. gracillima* only in the subgenus-level analyses.

All plants were maintained under standard glasshouse conditions with supplemental lighting in the research glasshouse at the University of Toronto Mississauga from autumn 2017 to summer 2018. We used the supplemental high-pressure sodium lighting (∼250 µmol m^−1^ s^−2^ at plant height) to maintain a minimum photoperiod of 14 h for the duration of the study. To control for the position of each *Cuscuta* plant and potential shading by the crown of its coleus host, we rotated pots regularly to vary the angle of light. Coleus hostas were kept well-watered until the *Cuscuta* began to flower, at which point we reduced watering frequency to encourage additional flowering and seed set.

For fluorescence imaging and pigment quantification (described below), we sampled three to five individuals per species for six organs or developmental stages (Fig. 1): (1) the apical 1 cm of free-living seedling (henceforth, seedling; sdlg); (2) young stem no more than 10 cm from the apical meristem (<5 cm for fluorescence imaging; young stem; y); (3) internodal stem sampled from between the third and fifth node past the young stem (old stem; o); (4) swollen portion of the stem attached to clusters of haustoria (haustorium; h); (5) whole flowers (flowers; f), which comprised a non-photosynthetic corolla and androecium, plus a photosynthetic calyx, gynoecium, receptacle and pedicel; and (6) immature seeds, dissected out of the developing fruit (seeds; sd). Though leaves in *Cuscuta* are vestigial, inconspicuous scales and were not sampled, we did measure *Ipomoea* leaves as an analogous baseline of optimal photosynthetic function.

### Fluorescence imaging

We took chlorophyll fluorescence measurements between 0945 and 1830 h on several days between 2 November 2017 and 24 August 2018 using an IMAGING-PAM M-Series, with the IMAG-MIN/GFP measuring head and IMAG-K6 camera (Heinz Walz GmbH, Effeltrich, Germany). Before taking measurements, we calibrated inhomogeneities of measuring sensitivity over the imaged area following the manufacturer's recommended image correction steps, i.e. using two layers of normal white printing paper at the fixed working distance of 7 cm. We brought images into focus using non-photosynthetically active near-infrared light, and manually drew sampling polygons using the manufacturer's ImagingWinGigE software.

To control for the position of each *Cuscuta* plant and potential shading by the crown of its coleus host, we sampled multiple replicate organs from each biological replicate (*Cuscuta +* coleus pair). For example, we conducted fluorescence imaging on four separate old stem samples from *Cuscuta monogyna* individual B. We first dark-acclimated *Cuscuta* and *Ipomoea* samples by covering them with an opaque black cloth for at least 30 min. Measuring light intensity was 0.2 µmol m^−2^ s^−1^ for all samples except the highly fluorescent *Ipomoea* leaf samples which we measured at half intensity (0.1 µmol m^−2^ s^−1^). We used a saturating light pulse with an intensity of 5200 µmol m^−2^ s^−1^ for a duration of 840 ms to determine *F*_o_ (minimum fluorescence) and *F*_m_ (maximum fluorescence; Lichtenthaler and Babani, 2004). Maximum quantum yield of open PSII centres (*F*_v_/*F*_m_) was derived following Genty *et al*. (1989):


$$\frac{F_{v}}{F_{m}}=\frac{F_{m}\;-\;F_{o}}{F_{m}}$$

After determining maximum quantum yield, samples were exposed to an actinic light with an intensity of 400 µmol m^−2^ s^−1^ for 5 min (occasionally up to 10 min if necessary for the fluorescence trace to reach steady state; Maxwell and Johnson, 2000), and then a second saturating pulse was applied to determine the maximal fluorescence of light-adapted tissues, *F*′_m_.

Immediately before each saturating pulse the steady-state fluorescence of light-adapted tissue, *F*_s_, was recorded. Using *F*_s_, *F*′_m_ and *F*_m_ we then calculated the effective quantum yield of a light-adapted sample (Φ_PSII_) which reflects the proportion of light absorbed by PSII used for photochemistry, as well as the proportion of light that is absorbed by PSII antenna and thermally dissipated via qE type xanthophyll-regulated non-photochemical quenching (Φ_NPQ_) using the following equations following Hendrickson *et al*. (2004):


$$\Phi_{\mathrm{PSII}}=1-\frac{F_{s}}{{F^{\prime }}_{m}}$$

$$\Phi_{\mathrm{NPQ}}=\frac{F_{s}}{{F^{\prime }}_{m}}-\frac{F_{s}}{F_{m}}$$

### Pigment quantification using HPLC

Immediately following fluorescence measurements, we flash froze each tissue sample in liquid nitrogen, and later ground them using a mortar and pestle. We extracted pigments from 40–50 mg aliquots of ground tissue into a total of either 1.25 or 2.05 mL solvent (98 % methanol and 2 % water buffered with 0.5 M ammonium acetate) for 2 h following the protocol developed by Junker and Ensminger (2016). Amber vials and dim ambient light were used to minimize light exposure and photobleaching.

We performed high-performance liquid chromatography (HPLC) analyses using an Agilent Infinity 1200 series Quaternary LC system (Agilent Technologies, Deutschland GmbH and Co. KG, Waldbronn, Germany) consisting of a quaternary pump, an autosampler set to 4 °C, a column compartment set to 25 °C, and a 1290 series photodiode array detector recording absorption at wavelengths of 290, 450 and 656 nm. Pigments were separated with a reversed-phase C_30_ column (5 µm, 250 × 4.6 mm; YMC Co. Ltd, Kyoto, Japan) using the protocol developed by Junker and Ensminger (2016). We detected peaks using ChemStation software and manual verification (Agilent Technologies, Santa Clara, CA, USA). Pigments were quantified using external standards.

We defined total chlorophyll content as the sum of chlorophyll (Chl) *a* and *b* concentration per gram tissue fresh weight (nmol g^−1^). Total carotenoid content was defined as the sum of violaxanthin, antheraxanthin, zeaxanthin, neoxanthin, lutein, α-carotene and β-carotene content. Although carotenoids are often normalized to Chl levels, this is not a meaningful ratio in *Cuscuta purpurata* because its Chl content approaches zero. Therefore, we normalized each carotenoid measured by total carotenoids. We calculated total xanthophylls by taking the sum of violaxanthin, antheraxanthin, zeaxanthin, neoxanthin and lutein. We calculated total xanthophyll cycle pigments (VAZ) as the sum of violaxanthin (V), antheraxanthin (A) and zeaxanthin (Z). We calculated the fraction of neoxanthin, violaxanthin and zeaxanthin (NVZ fraction; see Supplementary Dataset S2) as a proportion of total carotenoids.

### Null-hypothesis tests comparing clades and ontogenetic stages

Prior to analyses, we screened fluorescence and pigment data for errors and outliers in two ways. First, we manually inspected imaging-PAM fluorescence data to ensure the selected region of interest included only the tissue of interest and that all values were physiologically plausible. Second, we screened for statistical outliers using a Cook's distance threshold of four (Cook, 1977; Kim and Storer, 1996) which resulted in the removal of 9 % of measurements (280 of 3027) from downstream analyses of Φ_PSII_, *F*_v_/*F*_m_ and Φ_NPQ_. To compare pigment composition and fluorescence data across tissues within each taxon, we performed non-parametric Kruskal–Wallis tests (Kruskal and Wallis, 1952). We selected this over an ANOVA due to non-normality of pigment and fluorescence data, as assessed by Shapiro–Wilk tests (Shapiro and Wilk, 1965), non-normal residuals and missing data for some tissue–taxon combinations. When we detected a significant main effect (*P* ≤ 0.05), we followed up with pairwise Dunn tests (Dunn, 1964) using the dunnTest() function of the FSA package in R (Ogle *et al*., 2025).

**Fig. 2.**
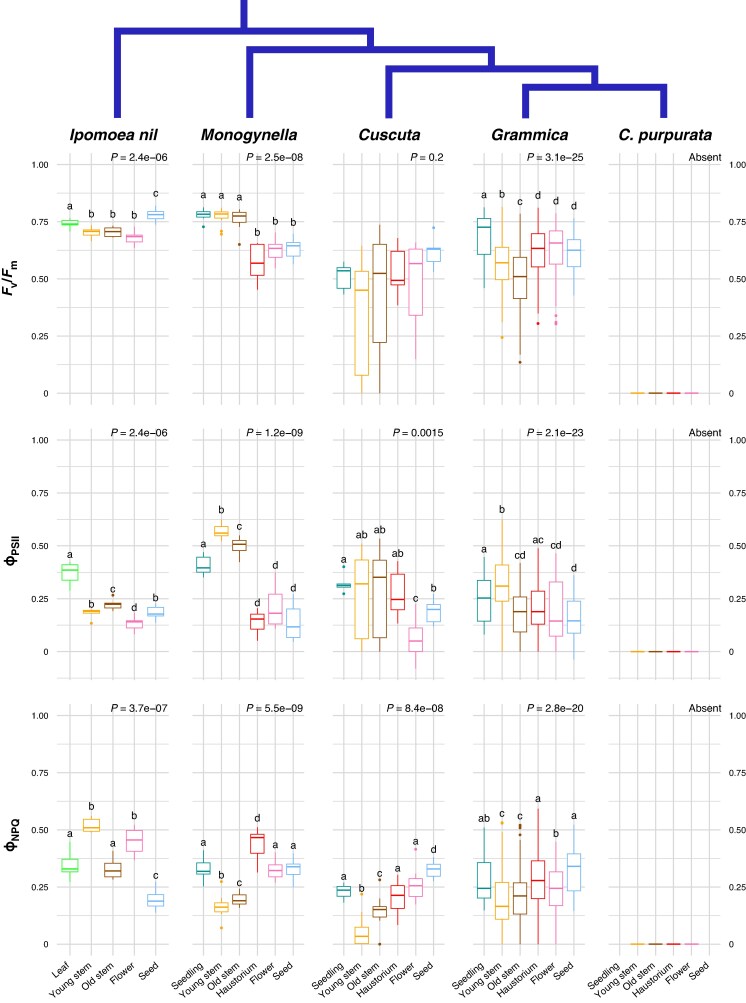
Variation in photosynthetic performance activity among clades and ontogenies of 14 *Cuscuta* species, grouped by clade. Leaves, old stems and young stems of the autotrophic relative *Ipomoea nil* are included for comparison, with phylogenetic relationships summarized in blue. Printed *P*-values represent the main effect of developmental stage using a Kruskal–Wallace test. Individual tissue means sharing the same letter within a single subplot are not significantly different (Dunn test *P* < 0.05, adjusted for multiple comparisons; *P*-values in Supplementary Data Appendix S5). Note the frequent appearance of a ‘U-shaped’ pattern with respect to ontogeny, particularly *F*_v_/*F*_m_ of subgen. *Grammica*, in which high parameter values appear early in vegetative growth (seedling and young stem) but decline with stem age, and then high levels appear again in reproductive tissue (seed). Ten species of subgen. *Grammica* are shown individually in Fig. S6. Pairwise comparisons of all means are shown in Appendix S3. Abbreviations: *F*_v_/*F*_m_ = maximum quantum yield of open PSII centres; Φ_PSII_ = effective quantum yield of PSII; Φ_NPQ_ = proportion of xanthophyll-regulated non-photochemical quenching.

**Fig. 3.**
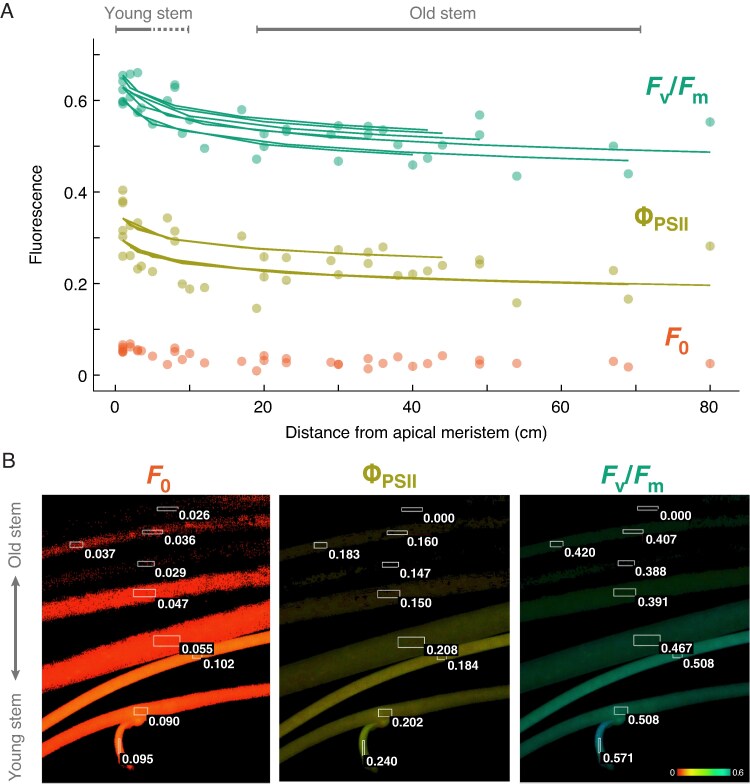
Ontogenetic loss of photosynthesis activity in stems of *Cuscuta* subgen*. Grammica* sect. *Oxycarpae*. (A) Hierarchical linear models support a significant decay of fluorescence-derived parameters *F*_v_/*F*_m_ (green; top) and Φ_PSII_ (yellow; middle) with distance from apical meristem in *C. cephalanthi* (Supplementary Data Table S1). Top grey lines illustrate regions of stem designated as ‘young’ or ‘old’ for comparative pigment and fluorescence analyses; see Methods for further detail. (B) Representative stem sample of the sister species *C. compacta* showing decreasing chlorophyll fluorescence (*F*_0_), Φ_PSII_ and *F*_v_/*F*_m_ across seven stem segments arranged from oldest (top) to youngest (bottom). Colour intensity and hue were determined by the parameter value at each pixel. Numbers indicate average parameter values within the rectangular areas of interest. Φ_NPQ_ was not significantly affected by stem age (Fig. S7).

In addition to the within-taxon analyses described above, we performed a second set of Kruskal–Wallis and Dunn tests to compare pigment and fluorescence data across tissues and across taxa simultaneously (Supplementary Data Appendices S3 and S4). All pairwise comparisons were adjusted using the Benjamini–Hochberg (BH) procedure (Benjamini and Hochberg, 1995) to control the false discovery rate (Jafari and Ansari-Pour, 2019). Analyses of fluorescence data without outlier removal did not affect the significance of the Kruskal–Wallis tests and influenced very few of the pairwise comparisons shown in Figs 1–3 (data not shown).

### Phylogenetic comparative analyses

To test whether loss of neoxanthin in *Cuscuta* evolutionary history was reversible (Collin and Miglietta, 2008), we performed an ancestral state estimation. First, we inferred the maximum likelihood phylogeny using RAxML-NG (Kozlov *et al*., 2019) and the model GTR+G+I from a gene-partitioned nucleotide sequence matrix consisting of previously published nuclear ribosomal 26S and chloroplast *rbc*L sequences (GenBank accessions in Table 1). 26S sequences were not available for *C. polygonorum*, *C. tasmanica* or *Ipomoea nil*, so we took a clade-based approach and substituted sequences from closely related taxa (*C. obtusiflora*, *C. tinctoria* and *I. lacunosa*, respectively). To improve branch length estimates, we also included sequences of *C. africana* (subgen. *Pachystigma*; GenBank accessions KJ436602 and KJ400037) despite lacking phenotypic data. Only the ribosomal 26S gene was used for the hybrid taxon *C. sandwichiana* due to conflicting plastid and nuclear gene trees. The resulting phylogeny was ultrametricized for downstream analyses by applying penalized likelihood with a correlated rate model implemented in the function chronos in R v.4.1.0 (R Core Team, 2020) using the packages phytools (Revell, 2012) and ape (Paradis and Schliep, 2019).

We scored neoxanthin ‘present’ in a taxon based on whether it had detectable neoxanthin in any life stage of any replicate. We estimated transition rates between presence and absence in a Bayesian phylogenetic framework using reversible-jump Markov chain Monte Carlo (RJ MCMC, Huelsenbeck *et al*., 2004) in RevBayes v.1.1.1 (Hohna *et al*., 2016) following Freund *et al*. (2018) and Lichter-Marck *et al*. (2020). In this model, the rate of loss was drawn from an exponential prior distribution with a mean of one expected character transition over the tree. Rates of gain were modelled as either zero (the irreversible model) or drawn from an exponential prior distribution with a mean of one expected character transition (the reversible model). We used RJ MCMC to sample between reversible and irreversible models, and posterior probabilities of each model were used to compare their relative fit. To ensure that model comparison was fair, we set the ancestral state at the root of the tree – that is, the most recent common ancestor (MRCA) of *Cuscuta* and *Ipomoea* – to be neoxanthin ‘present’ by fixing the root frequency such that the prior probability of neoxanthin was 1 (Goldberg and Igić, 2008). We performed four replicate MCMCs, each with 5000 generations with a burn-in of 1000 generations. Ancestral states for each node were sampled each generation; sampling the ancestral states during the RJ MCMC ensures that they include uncertainty about the transition model. We inspected the results of each individual run and the combined runs in R in the packages RevGadgets (Tribble *et al*., 2021) and coda (Plummer *et al*., 2006), first to ensure that chains had converged to the same posterior distribution, and second that an effective sample size of at least 650 was reached (Fabreti and Höhna, 2022). We compiled and visualized model-averaged transition rates and ancestral states on the tree using RevGadgets. Finally, we assessed prior sensitivity to verify that results were not strongly affected by our prior expectations. To do this, we repeated the ancestral state estimation while adjusting the expected number of changes on the tree (gain or loss of neoxanthin) to 2, 5 and 10.

To test if increased reliance on the lutein epoxide cycle in *Cuscuta* affects photosynthetic performance, we estimated evolutionary correlation between lutein epoxide concentration and Φ_PSII_ across the *Cuscuta* phylogeny in a Bayesian phylogenetic framework using a model of multivariate Brownian motion (Huelsenbeck and Rannala, 2003; May and Moore, 2020; May, 2022) implemented in RevBayes. We estimated correlations for each of the six *Cuscuta* ontogenetic stages assayed: seedling, young stem, old stem, haustorium, flower and seed. We did not include *Ipomoea* in these analyses due to differences in homology, and we also omitted the hybrid taxon *C. sandwichiana* on the premise that the reticulate evolutionary history of both the nuclear and chloroplast genomes probably contribute to Φ_PSII_. To perform this test, we fit a multivariate Brownian motion model to the data for each ontogenetic stage on the penalized likelihood chronogram (after dropping tips that did not have data for that stage) using two replicate MCMC analyses, each with 50 000 generations of 11 MCMC moves randomly chosen from four options, following a burn-in of 1000 generations. We performed MCMC diagnosis in the same way as the neoxanthin analysis (above). We assessed the significance of positive and negative correlation Bayes factors (Kass and Raftery, 1995; Huelsenbeck and Rannala, 2003), where the Bayes factor for a positive correlation ($BF^{+}$) was calculated as:


$$BF^{+}=\frac{\left(\frac{P[r\;>\;0\;|\;Y]}{P[r\leq 0\;|\;Y]}\right)}{\left(\frac{P[r\;>\;0]}{P[r\leq 0]}\right)}$$

in which P[r>0|Y] is the posterior probability of a positive correlation (i.e. the fraction of MCMC samples with a correlation >0), and P[r>0] is the prior probability of a positive correlation (and likewise for a negative correlation). The prior on the correlation parameter was symmetrical around zero, so the second ratio was set to 1. We determined the significance of each correlation by comparing two times the log Bayes factor of the positive correlation model (2lnBF^+^; Kass and Raftery, 1995). Support for a positive correlation (or negative, depending on the sign) increases with the magnitude of 2lnBF. In the same way, we tested correlations between lutein epoxide and *F*_v_/*F*_m_ to further test the relationship between lutein epoxide and photosynthetic performance, and lutein versus β-carotene concentrations to explore potential tradeoffs in carotenoid production.

The following R packages were also used for data analysis and visualization: colorBlindness (Ou, 2021), data.table v.1.16.4 (Barrett *et al*., 2024), dplyr (Wickham *et al.*, 2022), ggplot2 (Wickham, 2016), gridtext (Wilke, 2020), gtools (Warnes *et al.*, 2022), openxlsx (Schauberger and Walker, 2025), patchwork (Pedersen, 2022), plyr (Wickham, 2011), QsRutils (Quensen, 2020), RevGadgets (Tribble *et al*., 2021), rstatix (Kassambara, 2022) and tidyverse (Wickham *et al*., 2019). Data and code are available at: (https://github.com/jenna-tb-ekwealor/Cuscuta_phylo_photosynthesis).

## RESULTS

### Photosynthetic activity

Fluorescence measurements revealed distinct effects of tissue type on photosynthetic activity in each clade (Fig. 2; Supplementary Dataset S3). No fluorescence signal was obtained from *Cuscuta purpurata*, even when the Imaging PAM measuring light was increased to its highest setting (1.1 µmol m^−2^ s^−1^). Other species from subgen. *Grammica* showed highest *F*_v_/*F*_m_ in non-attached (free-living) seedlings, and the highest Φ_PSII_ in young stem tips, but both parameters declined with stem age and were at intermediate levels in haustorium, flower and developing seed tissues (Fig. 2; Supplementary Data Fig. S6). In subgen. *Monogynella* these three organs showed lower *F*_v_/*F*_m_ and Φ_PSII_ than all three stem tissues (seedling, young and old; *P* < 0.02; Appendix S3). In *C. cephalanthi* (subgen. *Grammica*) most of this decline in *F*_v_/*F*_m_ and Φ_PSII_ occurred in the distal (youngest) 10 cm of stem tissue (Fig. 3), but Φ_NPQ_ did not clearly change with stem age (*R*^2^ = 0.06, Table S1, Fig. S7), consistent with results across subgen. *Grammica* (Fig. S6) and the overall variable effects of clade and tissue (Fig. 2).

Leaves of the autotroph *Ipomoea* had higher *F*_v_/*F*_m_ than most tissues of the mixotrophic genus *Cuscuta*, but were comparable to stem tissues of *Cuscuta* subgen. *Monogynella* (the sister lineage to the rest of *Cuscuta*) and autotrophic seedlings of some species of subgen. *Grammica* (Fig. 2; Supplementary Data Appendix S3). Although the average Φ_PSII_ appears surprisingly higher in young and old stems of subgen. *Monogynella* (Fig. 2) and young stems of some species of subgen. *Grammica* (Fig. S6) compared to the leaves of autotrophic *Ipomoea* leaves, these differences were not significant (*P* = 0.24 and 0.34, respectively for *Monogynella*; Appendix S3). Most tissues in species from the phylogenetically nested subgen. *Grammica* had lower Φ_PSII_ than *Ipomoea* leaves or subgen. *Monogynella* stem tissue.

### Pigment composition

**Fig. 4.**
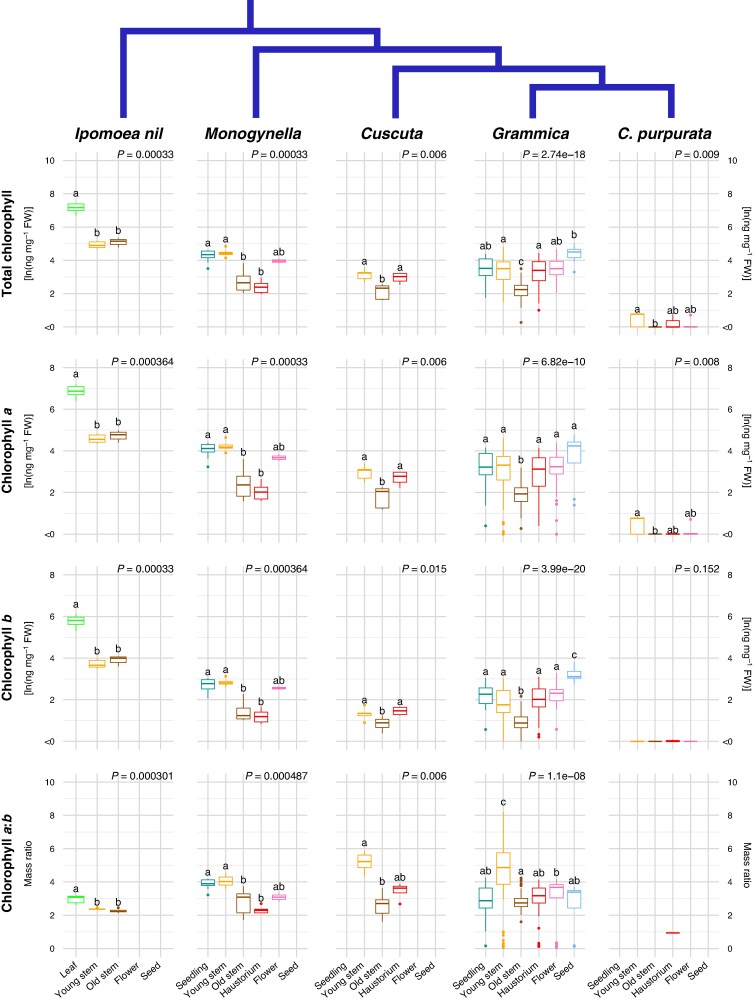
Variation in chlorophyll content by fresh weight (FW) and ontogenetic stages of 14 *Cuscuta* species, grouped by clade. Leaves, old stems and young stems of the autotrophic relative *Ipomoea nil* are included for comparison. Printed *P*-values represent the main effect of developmental stage using a Kruskal–Wallace test. Individual tissue means sharing the same letter within a single subplot are not significantly different (Dunn test *P* < 0.05, adjusted for multiple comparisons; *P*-values in Supplementary Data Appendix S6). For comparisons across taxa see Appendix S4.

**Fig. 5.**
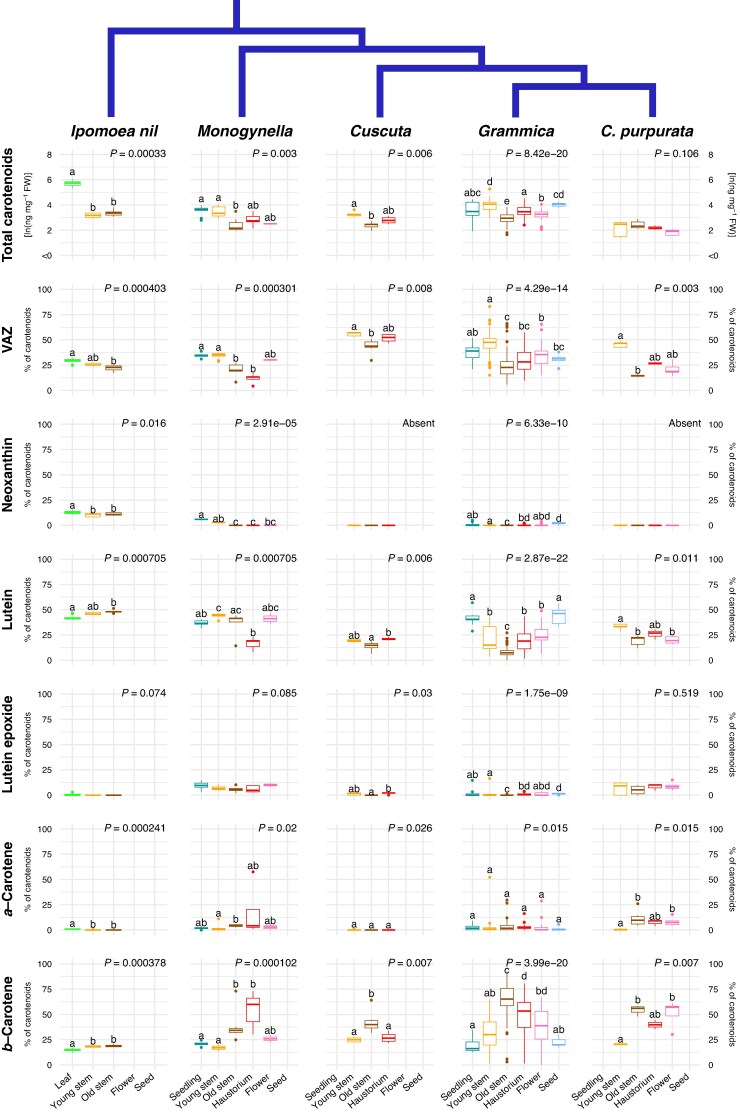
Variation in carotenoid content by fresh weight (FW) for total carotenoids and by percentage of total carotenoids for others, among clades and ontogenetic stages of 14 *Cuscuta* species grouped by clade. Leaves, old stems and young stems of the autotrophic relative *Ipomoea nil* are included for comparison. Printed *P*-values represent the main effect of developmental stage using a Kruskal–Wallace test. Individual tissue means sharing the same letter within a single subplot are not significantly different (Dunn test *P* < 0.05, adjusted for multiple comparisons; *P*-values in Supplementary Data Appendix S6). For comparisons across taxa see Appendix S4.

Chlorophyll and carotenoid content of fresh tissues differed significantly across developmental stages (Figs 4 and 5; Supplementary Data Appendix S4; pigment content ∼ ontogeny *H* = 38.7, *P* << 0.001), and major clades of *Cuscuta* (pigment content ∼ clade *P* < 0.01 for each pigment and pigment ratio; Appendix S4). *Cuscuta* tissues had generally less chlorophyll than *Ipomoea* stems, and several orders of magnitude less chlorophyll and carotenoids than *Ipomoea* leaves (Figs 4 and 5).

*Cuscuta purpurata*, the putatively non-photosynthetic species, lacked chlorophyll *b* and showed very low levels of chlorophyll *a* only in the young stem (Fig. 4). Among photosynthetic *Cuscuta* taxa, seedling and young stems had similar levels of chlorophylls *a* and *b* (Fig. 4), and most carotenoids (Fig. 5). Regardless of clade, chlorophyll content by fresh weight was lower in old stems than in young stems or seedlings, but similar to or occasionally higher than in young stems and flowers (Fig. 4; Supplementary Data Appendices S2 and S4). In the two species of subgen. *Grammica* for which we were able to sample developing seeds, chlorophyll *a* and chlorophyll *b* were most concentrated in these tissues.

In contrast to the consistent patterns of chlorophyll content in tissues across taxa, the relative chlorophyll content of the haustorium differed (Fig. 4). In subgen. *Monogynella*, chlorophyll content was lowest, comparable to that of an old stem. However, in subgen. *Cuscuta* and most species of subgen. *Grammica*, the chlorophyll content of the haustorium was significantly higher than that of old stems, instead comparable to the levels found in young stems (Fig. 4; Supplementary Data Appendix S4, Fig. S2). The ratio of chlorophyll *a* to *b* was typically highest in young stems compared to other tissues, and well being as higher in developing seeds of *C. cepthalanthii* (Fig. 4; Fig. S2).

Carotenoid content was broadly more similar across tissues and taxa (Fig. 5). Lutein and β-carotene were the two most abundant, together making up 50–75% of total carotenoids. Also among the most variable across tissues lutein was lowest in old stems and highest in developing seeds in subgenera *Cuscuta* and *Grammica*, whereas β-carotene showed the inverse pattern. It was lowest in seedlings and developing seeds, and most concentrated where lutein levels were at a minimum. At evolutionary scales, the concentrations of these traits relative to fresh weight were also negatively correlated in seedlings and young stems (Supplementary Data Fig. S5). We were not able to identify a large unknown peak visible at 290 nm, eluted at 11.9 s in HPLC chromatograms from taxa of subgen. *Monogynella* (data not shown).

**Fig. 6.**
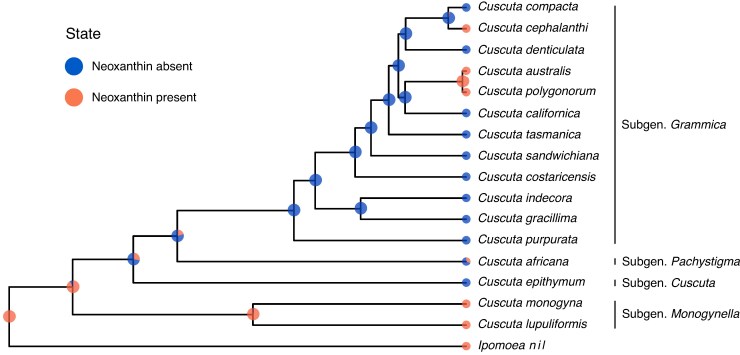
Ancestral state estimation of neoxanthin loss and gain in the 16 *Cuscuta* species used in this study. Tip-states were assigned based on pigment chromatography results (Fig. 5; Supplementary Data Fig. S3), except for *Cuscuta africana*, which was not sampled and therefore inferred by the analysis. Coloured circles at each node indicate the posterior probability of neoxanthin presence in any tissue (orange) or absence in all tissues (blue).

Also notable, neoxanthin was absent from all tissues of subgen. *Cuscuta*, eight species of subgen. *Grammica* (including holoparasitic *C. pupurata*) and certain older tissues of other species (Fig. 5; Supplementary Data Fig. S3; Appendix S2). When considering the relationships among these taxa, we found overwhelming support for a reversible model of neoxanthin evolution allowing regain of this trait, over the loss-only model predicted by Dollo's Law (posterior probability, PP = 0.999, Fig. 4). With a prior expectation of a single transition, we found that the MRCA of all *Cuscuta* probably produced neoxanthin (PP = 0.91). However, production for this carotenoid was probably absent in the MRCA of subgen. *Grammica*, *Pachystigma* and *Cuscuta* (PP = 0.72) and therefore lost after the evolution of parasitism and after the divergence of subgen. *Monogynella*. More recently, we inferred two re-gains of neoxanthin production: one at the MRCA of *C. polygonorum* and *C. australis*, and one in *C. cephalanthi* (PP > 0.97; Fig. 6). Finally, our model predicted that *Cuscuta* subgen. *Pachystigma* (represented by the taxon *C. africana*) does not produce neoxanthin (PP = 0.69). Sensitivity analyses revealed that the posterior probability of ancestral states at deeper nodes somewhat depended on the prior assigned to average number of transitions across the tree, although the maximum a posteriori reconstruction did not qualitatively change for *n* ≤ 10 (Fig. S4).

**Fig. 7.**
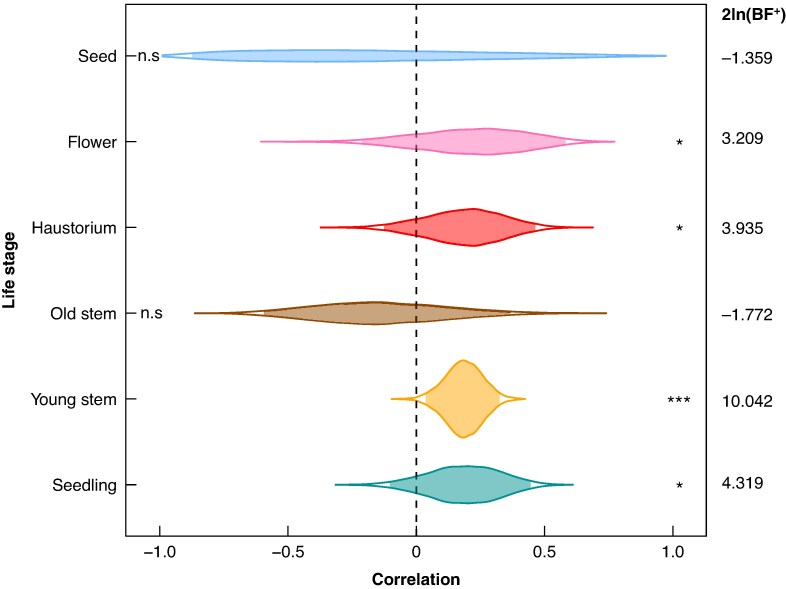
Bayesian phylogenetic correlations between photosystem II efficiency (Φ_PSII_) and lutein epoxide concentration in six ontogenetic stages (seedling, young stem, old stem, haustorium, flower and seed) of *Cuscuta* (*n* = 14 species). Violin plots represent posterior distributions of correlation coefficients estimated using a Brownian motion model of trait evolution. The shaded area within each violin represents the 95 % credible interval, and the dashed line represents 0 correlation. Asterisks indicate correlations significantly different from zero, assessed by Bayes factors: n.s., |2ln(BF^+^)| ≤ 2; *, 2 < |2ln(BF^+^)| ≤ 6; ***, 6 < |2ln(BF^+^)| < 10. Caution is warranted in interpreting the seed data, which only included samples from three species (*C. australis*, *C. cephalanthi* and *C. polygonorum*).

When controlling for phylogeny, lutein epoxide concentration and Φ_PSII_ were significantly positively correlated in seedling, haustorium, young stem, and flower tissues [2ln(BF^+^) > 2; Fig. 7]. By contrast, we found no significant correlation in old stem or seed tissue. *F*_v_/*F*_m_ and lutein epoxide concentrations coevolved differently: while these pigment concentrations were also positively correlated across the phylogeny in flowers, young stems and seedlings, they were not correlated in haustorium, and were negatively correlated old stem tissue, and developing seeds of the three taxa in subgen *Grammica* that could be measured (*C. australis*, *C. cephalanthi* and *C. polygonorum*; Supplementary Data Fig. S5).

## DISCUSSION

In this study we demonstrate that photosynthesis in *Cuscuta* is not simply a vestigial trait but is actively regulated across developmental stages. Rather than being uniformly suppressed in favour of its parasitic lifestyle, photosynthetic activity, chlorophyll content and carotenoid concentrations vary predictably with energetic demands at different life stages. *Cuscuta* photosynthesis is highest in tissues that are in active growth or development, such as non-attached seedlings and shoot tips. It then declines in older vegetative structures, and increases again in reproductive tissues, especially as developing seeds store carbohydrates. This results in a ‘U-shaped’ pattern with respect to ontogeny (Figs 2, 4 and 5) that mirrors variation in metabolic demands and energy investments across the organism. Such dynamic regulation suggests the possibility that photostasis, the energy balance between photosynthesis as the source of energy and metabolic sinks (Paul and Foyer, 2001; Falkowski and Chen, 2003), probably drives both tissue-specific plasticity and selection, despite the ability to acquire carbon from its hosts. While this study centres on *Cuscuta*, such facultative photosynthesis is by no means unique. For example, the independently evolved stem parasite *Arceuthobium* M.Bieb has extensive achlorophyllous endophytic structures, but also photosynthetic exophytic shoots that bear flowers and fruits and, depending on the species, assimilate up to 50 % of the plant's carbon (Rey *et al*., 1991; Těšitel, 2016).

### Phylogenetic and ontogenetic variation in photosynthetic function

Most species in *Cuscuta* subgen. *Grammica* exhibit the ‘U-shaped’ pattern of photosynthetic activity (*F*_v_/*F*_m_ and Φ_PSII_), with higher values in young and rapidly growing tissues, lower activity in old stems, and intermediate levels in haustorium and floral tissue of some species (Fig. 2; Supplementary Data Fig. S6). However, this pattern is not fully consistent even among species of subgen. *Grammica* (Fig. S6), let alone across *Cuscuta* as a whole clade (Fig. 2), indicating an interaction between phylogeny and ontogeny. The most striking exception can be seen in the holoparasite *C. purpurata* (also from subgen. *Grammica*), which completely lacks photosynthetic function at any life stage (Fig. 2), as evidenced by undetectable or negligible levels of chlorophyll (Fig. 4), and complete lack of photosynthetic genes (Braukmann *et al*., 2013; Banerjee and Stefanović, 2023).

In contrast, species in subgen. *Monogynella* showed high photosynthetic activity in vegetative stages – comparable to the leaves of its autotrophic relative *Ipomoea* – but also a marked drop in activity in haustorium, flower and developing seeds (Fig. 2; Supplementary Data Appendix S3). The retention of a relatively complete plastome in subgen. *Monogynella*, along with low levels of carbon assimilation, and more modest ultrastructure simplification of the chloroplast suggest an evolutionary trajectory in this group distinct from that in subgen. *Grammica*, where species have substantial reductions in genome content and plastid structure, including 15 % of photosynthesis-related orthologues in the relatively functional *C. australis* to complete loss of autotrophy in *C. purpurata* (Fig. 1; Machado and Zetsche, 1990; Braukmann *et al*., 2013; Sun *et al*., 2018; Vogel *et al*., 2018; Banerjee and Stefanović, 2023). In summary, *Cuscuta* subgen. *Monogynella*, the physiology of which has been the most intensely studied to date (Machado and Zetsche, 1990; Choudhury and Sahu, 1999; Snyder *et al*., 2004, 2005; Qin *et al.*, 2008), appears to have the most intact and effective photosynthetic machinery among *Cuscuta* species, but even in this case is mostly recycling respired carbon within the plant body than assimilating it from the environment (Hibberd *et al*., 1998). At the same time, hemiparasitic (so-called ‘cryptically photosynthetic’) species within subgen. *Grammica* show little evidence of differential photosynthetic efficiency (Figs S2, S3 and S6), just as plastome gene content is similar within this clade (Braukmann *et al*., 2013; Banerjee and Stefanović, 2023).

### Carotenoid dynamics at organismal and evolutionary scales

Carotenoids play a pivotal role in light capture and preventing photooxidative damage (Simkin *et al*., 2022). Carotenoid profiles can therefore reveal how plants balance light harvesting and photoprotection to improve photosynthetic efficiency and provide clues to the dynamic regulation of photosynthesis in *Cuscuta*. At the organismal level, differences in *F*_v_/*F*_m_ across tissues in subgenus *Grammica* correspond quite nicely with the ‘U’ shape seen in lutein and total chlorophyll (Figs 2, 4 and 5). Thus, when quantum yield is high lutein contributes to a well-oiled antenna system that delivers excitation to the core of PSII where photochemical quenching takes place. By contrast, β-carotene shows the reverse relationship (Fig. 5). β-carotene can both support electron transfer in the reaction centre and quench excitation from triplet chlorophyll in photosystem I, thereby protecting photosystem II in situations where energy transfer is hampered. Thus, it may be that in mature stems where photosynthesis is low anyway, increased β-carotene contributes to increased photoprotection (Jahns and Holzwarth, 2012). At the evolutionary level, we also found support for a trade off between lutein and β-carotene production in seedling and young stem tissues, specifically, in the strong negative correlation observed across the phylogeny (Supplementary Data Fig. S5). Together, these two compounds comprise 50–75% of the total carotenoids across species and tissues (Fig. 5).

In *Cuscuta*, lutein is also involved in the photoprotective lutein epoxide (LxL) cycle as a product of the light-driven de-epoxidation of lutein epoxide (Bungard *et al*., 1999; Leonelli *et al*., 2017). In our study, concentrations of lutein epoxide were positively correlated with photosynthetic efficiency (Φ_PSII_), in flowers, haustoria, seedlings and, particularly, young stems across *Cuscuta* (Fig. 7). This is consistent with the hypothesized role of lutein epoxide in light-harvesting efficiency under limiting light (García Plazaola *et al*., 2007; Kruk and Szymanska, 2008; but see Bungard *et al*., 1999). In short, the light-harvesting role of lutein epoxide seems to play an enhanced role in life stages that have higher energetic needs, while functioning in some other way, like for photoprotection under sudden irradiance changes (García Plazaola *et al*., 2007; Esteban *et al*., 2009) or as seen in this study, in less photosynthetic regions such as haustoria and older stems.

Our findings also provide an evolutionary context for the unique violaxanthin cycle reported in *Cuscuta.* While this xanthophyll cycle is almost universally conserved across vascular plants, in some *Cuscuta* species 9-*cis* violaxanthin has completely replaced neoxanthin in the light-harvesting complex (Bungard *et al*., 1999; Snyder *et al*., 2004). Extending these findings, we did not detect neoxanthin in any life stage of any species in subgen. *Cuscuta* nor in eight species of subgen. *Grammica* (Fig. 5). Given the phylogenetic relationships of these species, we find strong evidence (PP = 1.00) that neoxanthin production was re-gained twice in *Grammica* after an initial loss shortly after the divergence from the clade containing subgen. *Monogynella* (Fig. 6). Closely related species within subgen. *Grammica*, namely *C. polygonorum* and *C. cephalanthi*, had detectable amounts of neoxanthin in some tissue types but not others (Supplementary Data Fig. S3), suggesting that phenotypic loss may be caused by a complex mosaic of factors, from changes in gene regulation to complete genetic loss or pseudogenization.

Evidence of phenotypic loss through gene regulation is also evident in subgen. *Monogynella*, the earliest diverging lineage of *Cuscuta*. For example, in *C. monogyna*, neoxanthin was detected in seedling and young stem, but not in old stem, haustorium or flower (Supplementary Data Appendix S2). Bungard *et al*. (1999) characterized neoxanthin loss and the unique xanthophyll cycle in *C. reflexa*, another species belonging to subgen. *Monogynella*, not sampled in our study. While these authors did not specify exactly what portion of the plants were used, it is most likely that neoxanthin was measured from stem tissue without distinguishing its age. Indeed, *C. reflexa* may also produce neoxanthin in seedlings and young stems like others in its subgenus (Fig. 5), but this prediction needs to be tested. If this were the case, it may not be that *C. reflexa* ‘lacks the synthesis step from violaxanthin to neoxanthin’ as was concluded by Bungard *et al*. (1999), but that instead neoxanthin is differentially expressed in different life stages, and in some lineages may have been suppressed before the total loss of neoxanthin synthesis ability.

Loss of neoxanthin in species or life stages that are photosynthetic may be compensated for by possible light-harvesting capabilities of lutein epoxide in *Cuscuta.* In fact, the widespread conservation of neoxanthin among land plants may instead be due to a role outside of light harvesting and photosynthesis (Bungard *et al*., 1999). Neoxanthin is a precursor for the plant growth regulator abscisic acid (ABA), which parasitic plants can acquire directly from their host through haustoria (Milborrow and Lee, 1997; Schwartz *et al*., 1997), or synthesize via alternative pathways (Qin *et al*., 2008). With an alternative photoprotective route through the lutein epoxide cycle and/or 9-*cis* violaxanthin, plus an alternative source of ABA, some lineages of *Cuscuta* may simply have lost the need for neoxanthin.

### Eco-evolutionary implications

Our findings underscore the need to study plants across both phylogeny and ontogeny, with a perspective that centres them in their full ecological context. The retention of chlorophyll (Fig. 4; Supplementary Data Fig. S2) and photosynthetic function (Figs 2 and 3) across the *Cuscuta* trophic and phylogenetic spectra (Fig. 1; Fig. S1), despite their dependence on host-derived carbon, suggests that the energetic needs of active growth and development are providing the selective pressure for retaining photosynthetic ability in most *Cuscuta* species, especially as independent (non-attached) seedlings. Indeed, photosynthesis-related genes appear to be under the highest selective constraint of any genes within the plastomes of *Cuscuta* (Banerjee and Stefanović, 2020, 2023). Even in obligately autotrophic *Ipomoea*, higher *F*_v_/*F*_m_ values are found in leaves and developing seeds than in photosynthetic stems (Fig. 2).

Carotenoid content also parallels these trends in chlorophyll content and photosynthetic activity across tissues (Figs 5 and 6; Supplementary Data Figs S3–S5). At least for lutein epoxide, its evolution across the genus is most strongly correlated with photosystem II performance at the rapidly growing shoot tips (Fig. 7; Supplementary Data Fig. S5). However, ontogeny–species–carotenoid relationships are more complex, given the photoprotective and other physiological roles of these compounds. For example, in non-photosynthetic *Cuscuta purpurata* most carotenoids are expressed at comparable levels to photosynthetic relatives, but neoxanthin has been lost or only expressed in some tissues across most of the genus (Figs 3, 5 and 6). Experiments comparing gene expression (transcriptomes) across developmental stages will probably provide additional insight into the proximal mechanisms underlying this regulation. Nonetheless, the benefits of continued regulation and expression of photosynthesis machinery and accessory compounds may help explain why, from an evolutionary perspective, only a substantial minority of parasitic plants have become entirely non-photosynthetic.

## Supplementary Material

mcaf145_Supplementary_Data

## Data Availability

Raw data are archived in Supplementary Data Appendices S1 and S2. All data are also archived on GitHub, along with additional code and analysis outputs: https://github.com/jenna-tb-ekwealor/Cuscuta_phylo_photosynthesis.
