# Supplementary material for: Photosynthetic activity in the heterotrophic plant genus Cuscuta (Convolvulaceae) is modulated by phylogeny and ontogeny
Source: Ann Bot. 2025 Jul 9;137(5):1187–202. doi: 10.1093/aob/mcaf145 (PMC13197604; doi:10.1093/aob/mcaf145)
Supplement: mcaf145_Supplementary_Data [file mcaf145_supplementary_data.zip › Supplementary_Table_and_Figures.pdf]

Supplement to Schneider et al. 2025 *Annals of Botany*

Photosynthetic activity in the heterotrophic plant genus *Cuscuta* (Convolvulaceae) is modulated by phylogeny and ontogeny

Adam C. Schneider, Jenna T. B. Ekwealor, Ariana Besik, Nurulain Ibrahim, Ingo Ensminger, and Saša Stefanović

Supplementary Tables and Figures:

Table S1: Estimated parameters from hierarchical linear models of the effect of *Cuscuta* stem age on F_v/F_m, Φ_{PSII}, and Φ_{NPQ}

Figure S1: Phylogenetic distribution of sampled taxa within *Cuscuta*.

Figure S2: Variation in chlorophyll content among species and ontogenies of *C.* sect. *Grammica*

Figure S3: Variation in carotenoid content among species and ontogenies of *C.* sect. *Grammica*

Figure S4: Sensitivity of neoxanthin Bayesian ancestral state estimates to the choice of prior.

Figure S5: Additional phylogenetically informed correlations in six ontogenetic stages.

Figure S6: Variation in F_v/F_m, Φ_{PSII}, and ΦNPQ among species and ontogenies of *C.* sect. *Grammica*

Figure S7: Evidence that Φ_{NPQ} of *Cuscuta* stem tissue did not significantly decay with age.

Appendices:

Available at Annals of Botany online. Complete descriptions are at the end of this document.

Appendix S1: Raw data and summary means from chlorophyll fluorescence imaging

Appendix S2: Raw data and summary means from HPLC pigment quantification

Appendix S3: *P*-values for all ontogeny-subgenus pairings of F_v/F_m, Φ_{PSII}, and Φ_{NPQ}

Appendix S4: *P*-values for all ontogeny-subgenus pairings of pigment concentrations

Appendix S5: *P*-values for post hoc tests shown in Fig. 2 (F_v/F_m, Φ_{PSII}, and Φ_{NPQ} by clade)

Appendix S6: *P*-values for post hoc tests shown in Figs. 4–5 (pigment concentration by clade)

Appendix S7: *P*-values for post hoc tests shown in Fig. S6 (F_v/F_m, Φ_{PSII}, and Φ_{NPQ} – *Grammica*)

Appendix S8: *P*-values for post hoc tests shown in Figures S2–S3 (pigments – *Grammica*)

Table S1. Estimated parameters from the hierarchical linear models $f \sim$ log(Distance.from.apical.meristem) + (1 | Genotype/Sample), in which seven individual stems from two different *Cuscuta* individual grown from seed were sampled at each internode (n=40). *** denotes P < 0.001 calculated using the R package *stargazer*. R^2 values calculated following Nakagawa and Schielzeth (2012).

Parameter	$\boldsymbol{F_v/F_m}$	$\boldsymbol{\Phi_{PSII}}$	$\boldsymbol{\Phi_{NPQ}}$
log(distance from AM)	–0.032 ± 0.004***	–0.023 ± 0.005***	–0.012 ± 0.007
Constant	0.63 ± 0.02***	0.32 ± 0.03***	0.31 ± 0.03***
Marginal R^2 (fixed effects only)	0.55	0.26	0.06
Conditional R^2 (full model)	0.74	0.58	0.28

Figure S1. Phylogenetic placement of species used for fluorescence imaging and pigment analysis shown on a summary evolutionary hypothesis for *Cuscuta* (dodders; Convolvulaceae). Phylogenetic tree adopted and modified from Costea *et al.* (2015). Numbers above branches indicate bootstrap support for labeled clades and backbone relationships as reported by García *et al.* (2014). Infrageneric classification (subgenera and sections) is indicated on the left; numbers in square brackets correspond to the number of species found in those groups (see Costea *et al.*, 2015). Fourteen *Cuscuta* species sampled in this study, evenly spanning the phylogeny of the genus, are indicated on the right; compare with Table 1 for voucher information, provenance of material, and further details

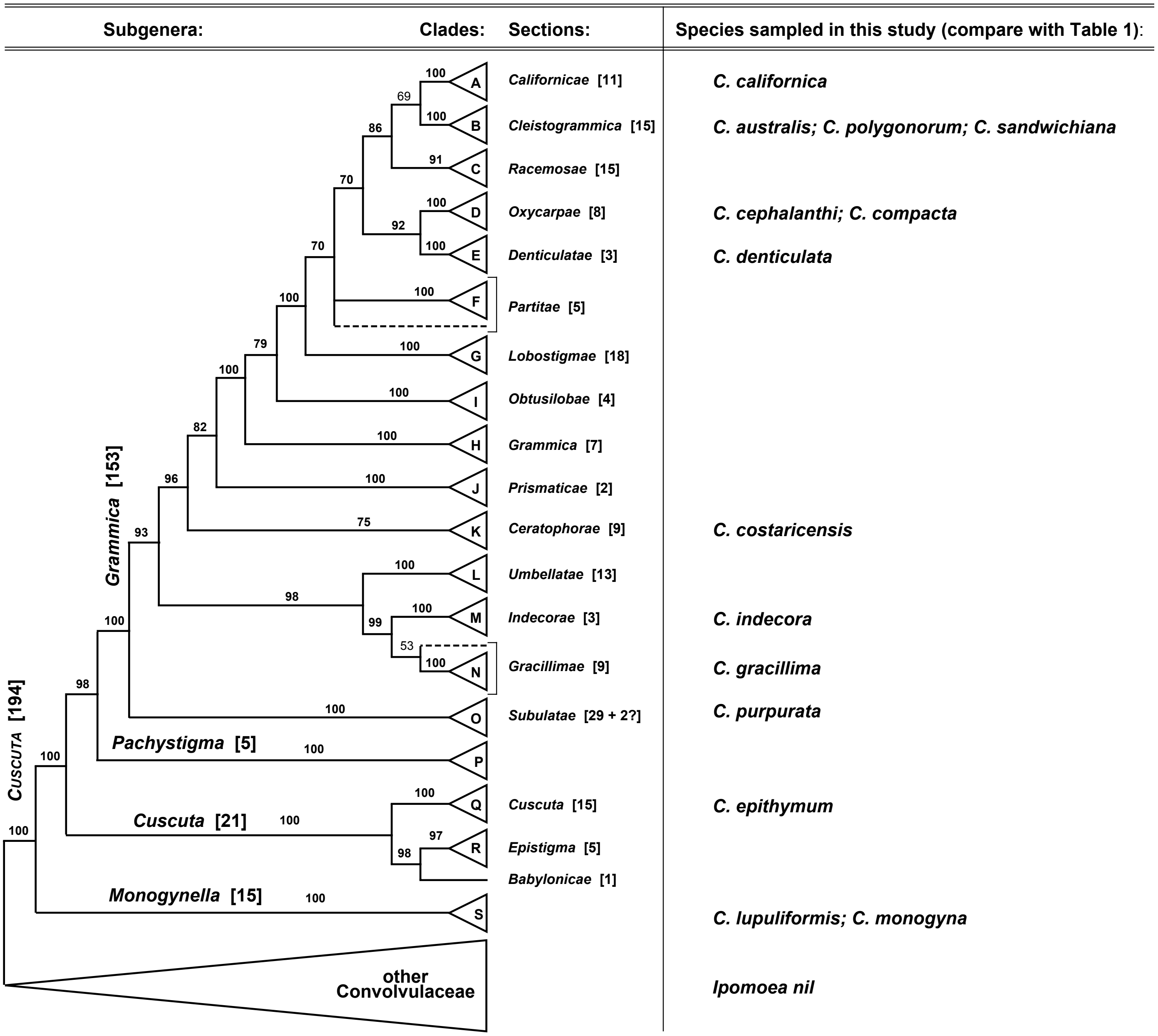

Figure S2. Variation in chlorophyll content among species and ontogenies of *Cuscuta* sect. *Grammica,* with phylogenetic relationships summarized in blue (after García *et al*., 2014). Plastid (pt) and nuclear (nu) relationships shown for the intersectional hybrid *Cuscuta sandwichiana*.

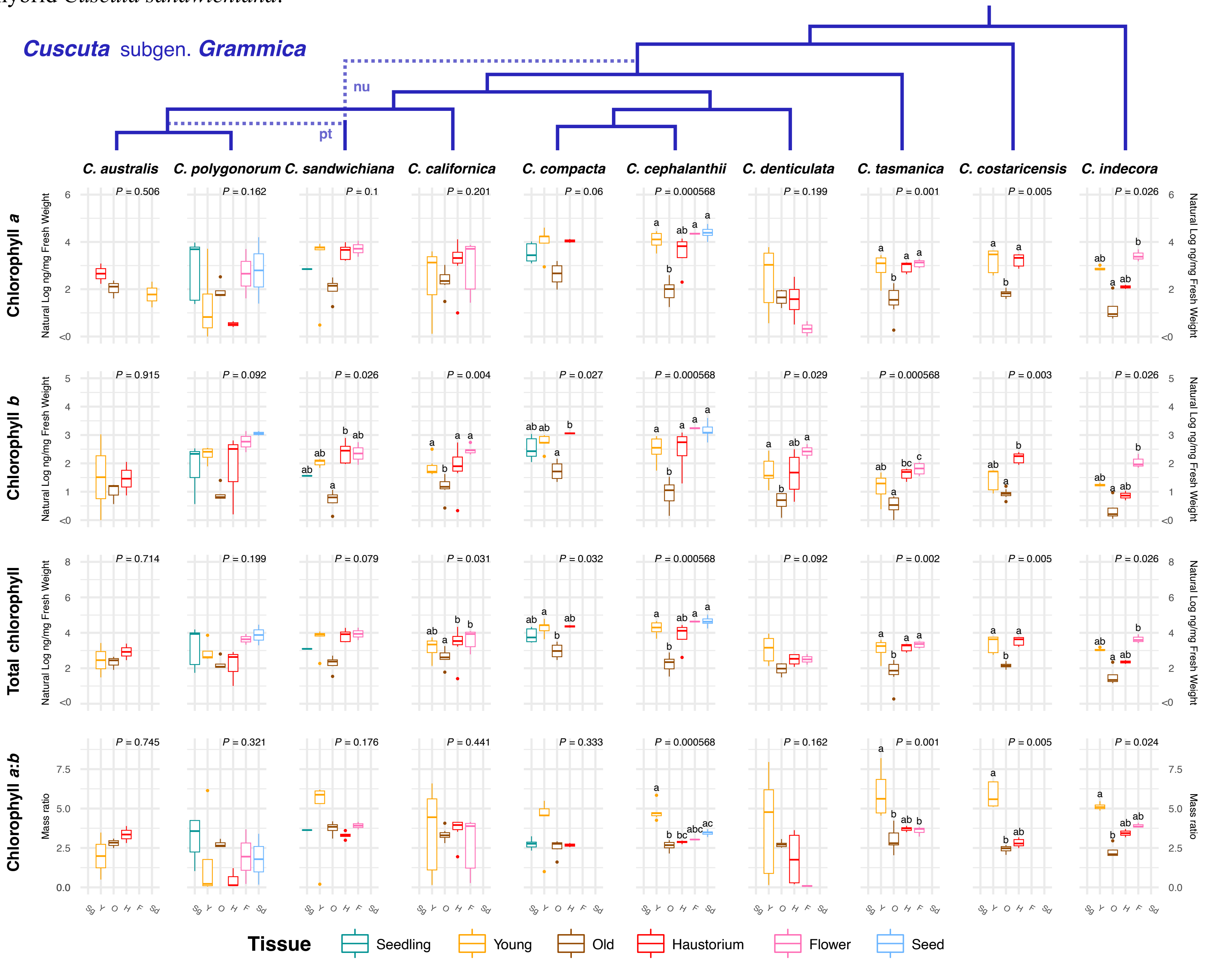

Figure S3. Variation in carotenoid content among species and ontogenies of ten species within *Cuscuta* subgen. *Grammica*. Phylogenetic relationships summarized in blue (after García *et al*., 2014). Plastid (pt) and nuclear (nu) relationships shown in dashed lines for the intersectional hybrid *Cuscuta sandwichiana*.

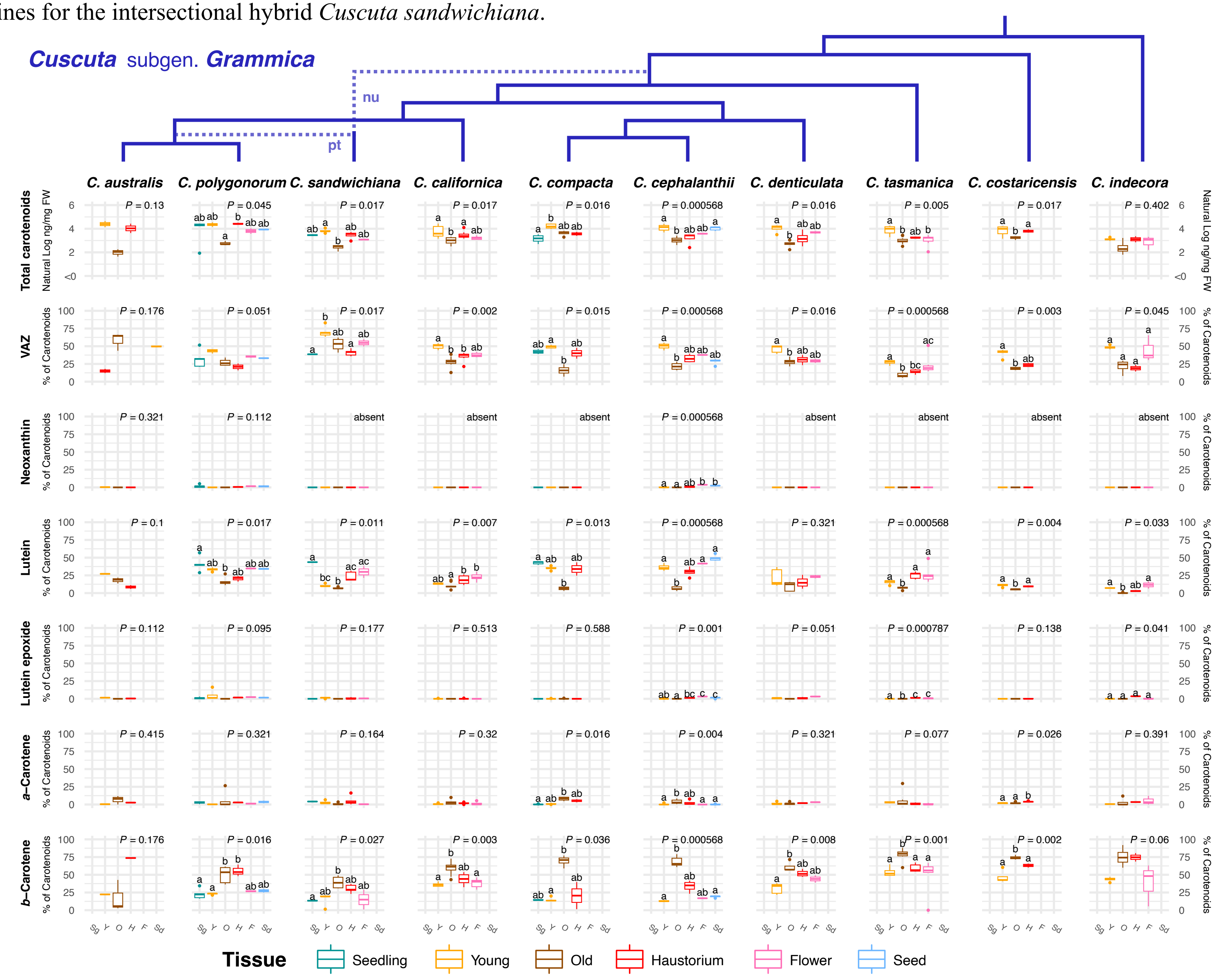

Figure S4. Sensitivity of neoxanthin ancestral state estimates to prior choice of (**A**) 2, (**B**) 5, and (**C**) 10 gains or losses of neoxanthin production. Tip-states were assigned based on HPLC results (Figs. 2, S3), except for *Cuscuta africana*, which was not sampled and had to be inferred by the analysis. Colored circles at each node indicate the posterior probability of neoxanthin presence in any tissue (orange) or absence in all tissues (blue).

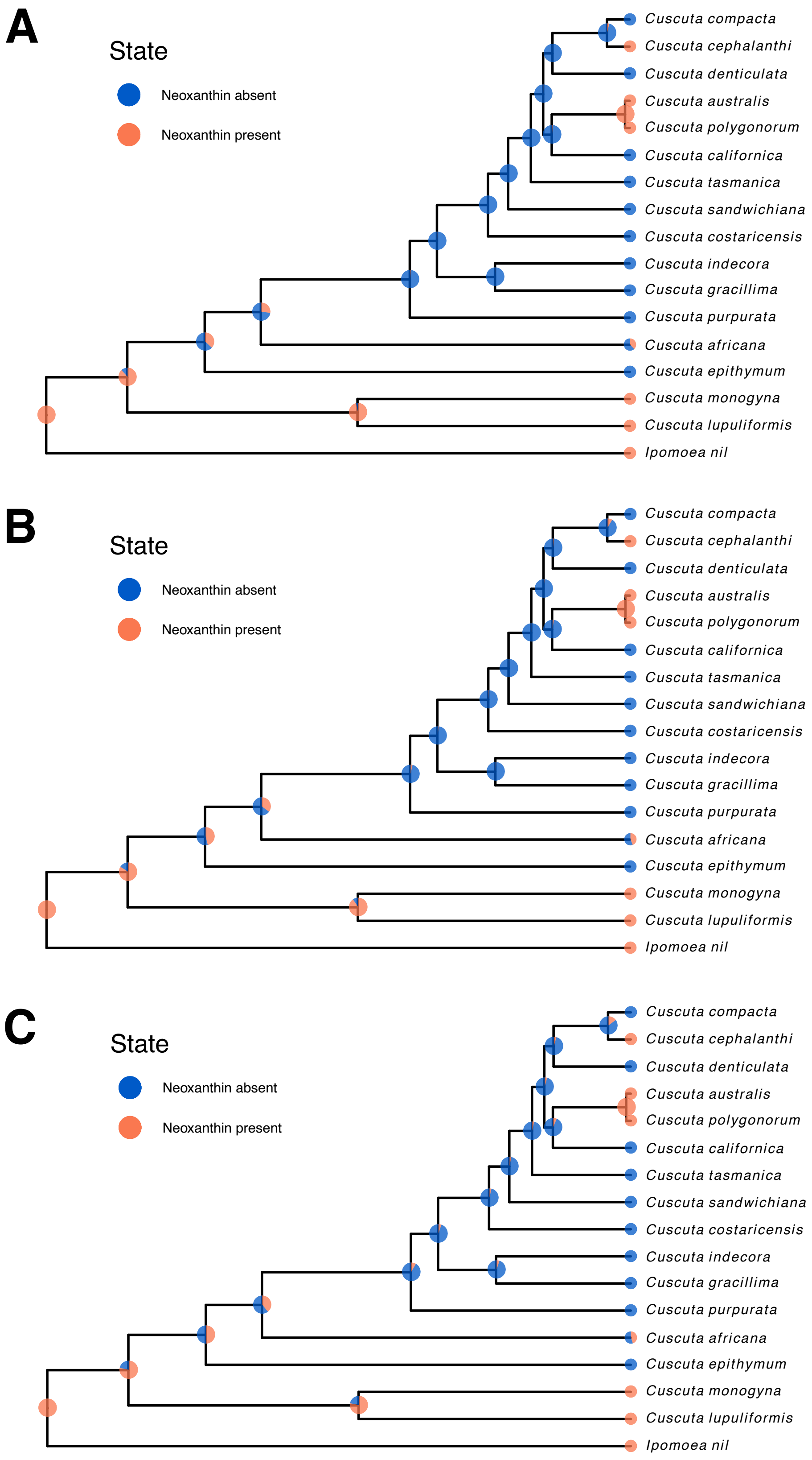

Figure S5. Bayesian phylogenetic correlations in six ontogenetic stages of *Cuscuta* (n = 14 species) between (**A**) lutein and beta-carotene (ng/mg fresh weight), and (**B**) maximum photosystem II efficiency (F_v/F_m) and lutein epoxide concentration. Violin plots represent posterior distributions of correlation coefficients estimated using Bayesian phylogenetic comparative methods (Brownian motion model). The shaded area within each violin represents the 95% credible interval, and the dashed line represents 0 correlation. Asterisks indicate correlations significantly different from zero, assessed by Bayes Factors (BF): (n.s. $|2\ln(BF^+)| \leq 2$, * $2 < |2\ln(BF^+)| \leq 6$, ** $6 < |2\ln(BF^+)| \leq 10$. Caution is warranted in interpreting the seed data, which only included samples from three species (*C. australis, C. cephalanthi,* and *C. polygonorum*).

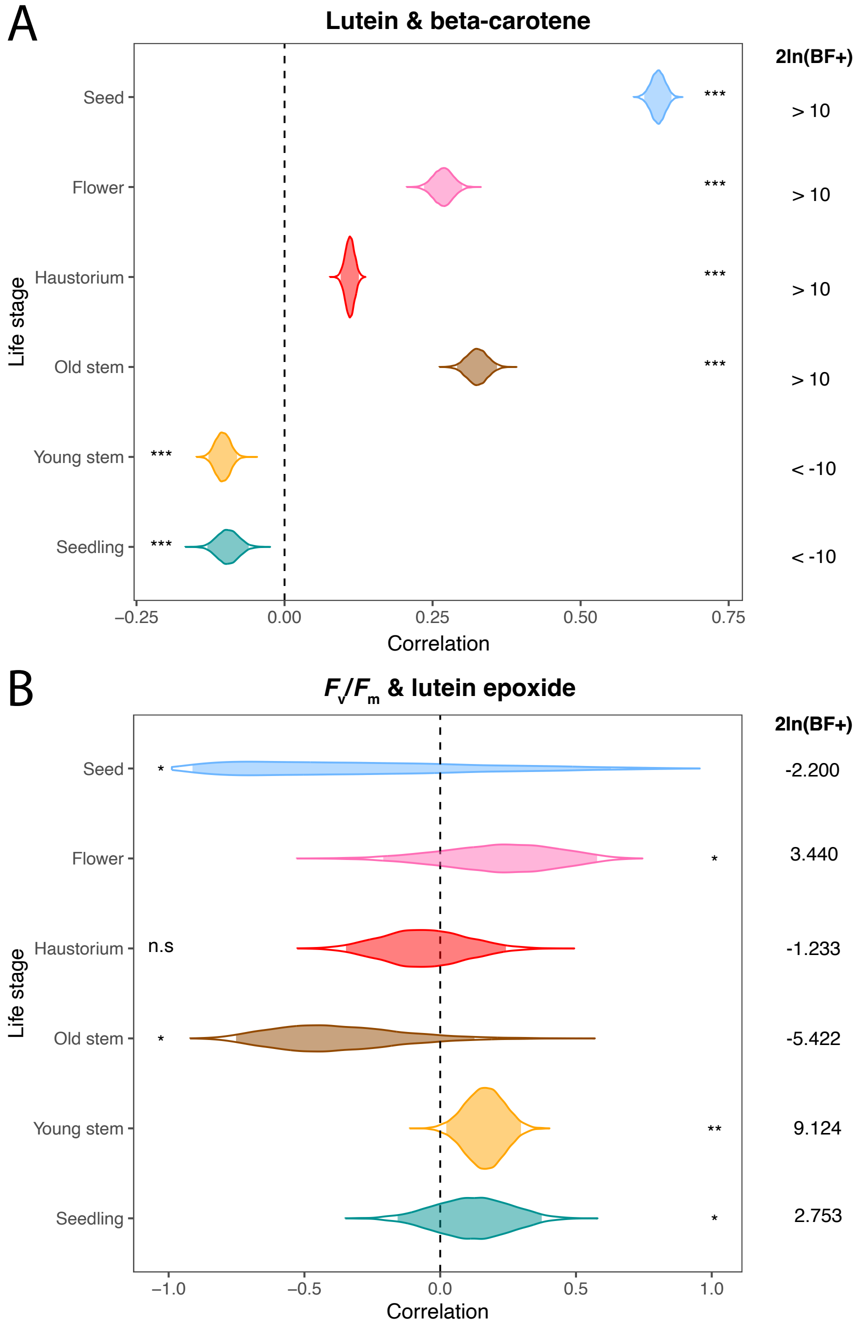

Figure S6. Variation in photosynthesis activity among species and ontogenies of *Cuscuta* subgen. *Grammica* (excluding *C. purpurata,* which is sister to the clade shown; compare with Fig. 2), with phylogenetic relationships summarized in blue (after García *et al*., 2014). Alternative plastid (pt) and nuclear (nu) relationships shown in dashed lines for the intersectional hybrid *Cuscuta sandwichiana*. Note in some cases (e.g., F_v/F_m of *C. cepthalanthi*, the appearance of a "U-shaped" pattern, in which high parameter values appear early in vegetative growth (seedling and young stem) but declining with stem age, then high levels again in reproductive tissue (seed).

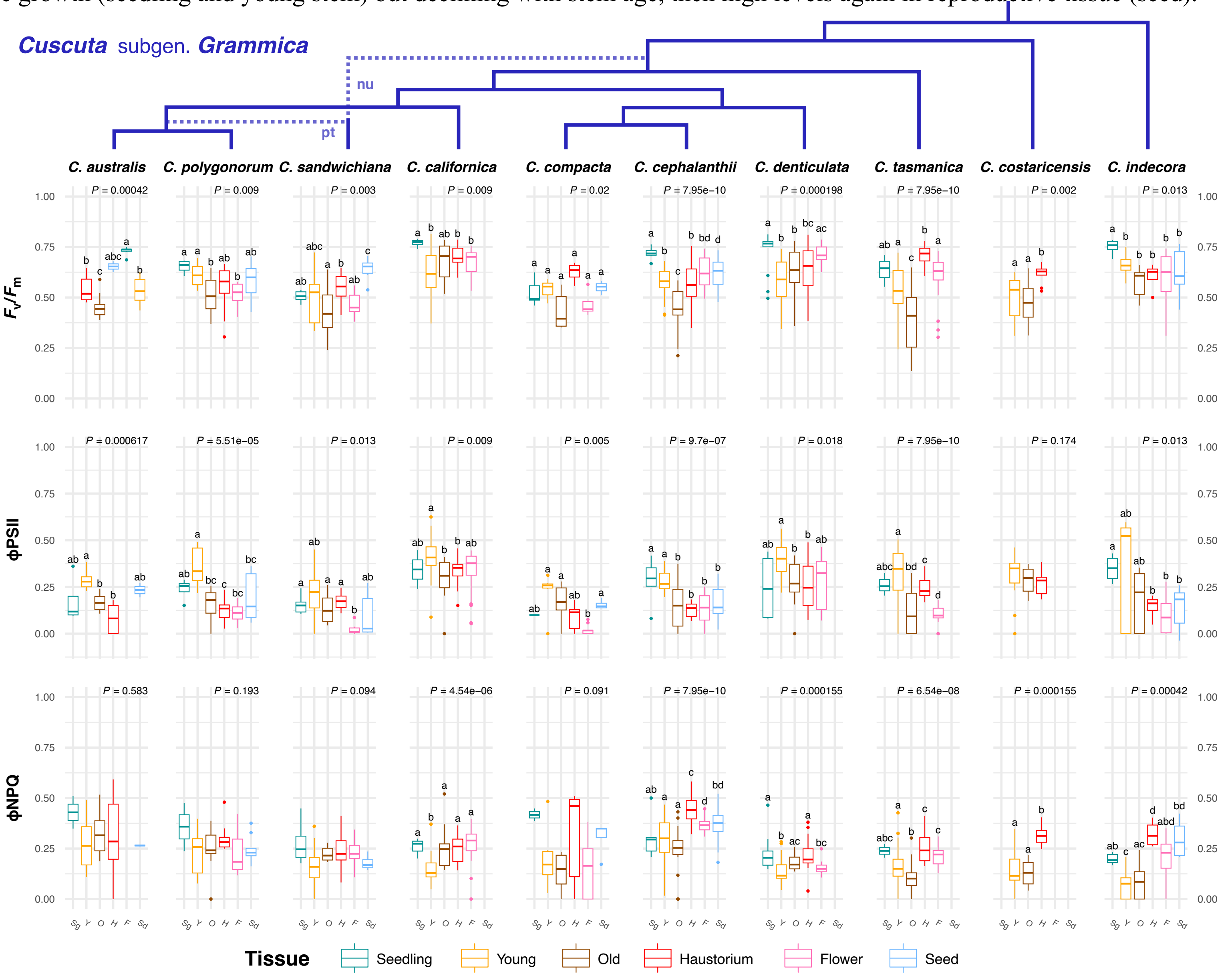

Figure S7. Φ_{NPQ} of *Cuscuta* stem tissue did not significantly decay with age. **(A)** Variation in Φ_{NPQ} by distance from apical meristem from seven stems and two individuals of *C. cephalanthi* (Table S1). Top gray lines illustrate regions of stem designated as "young" or "old" for comparative pigment and fluorescence analyses; see methods for further detail. **(B)** Representative segments of a single *C. compacta* stem arranged by age.

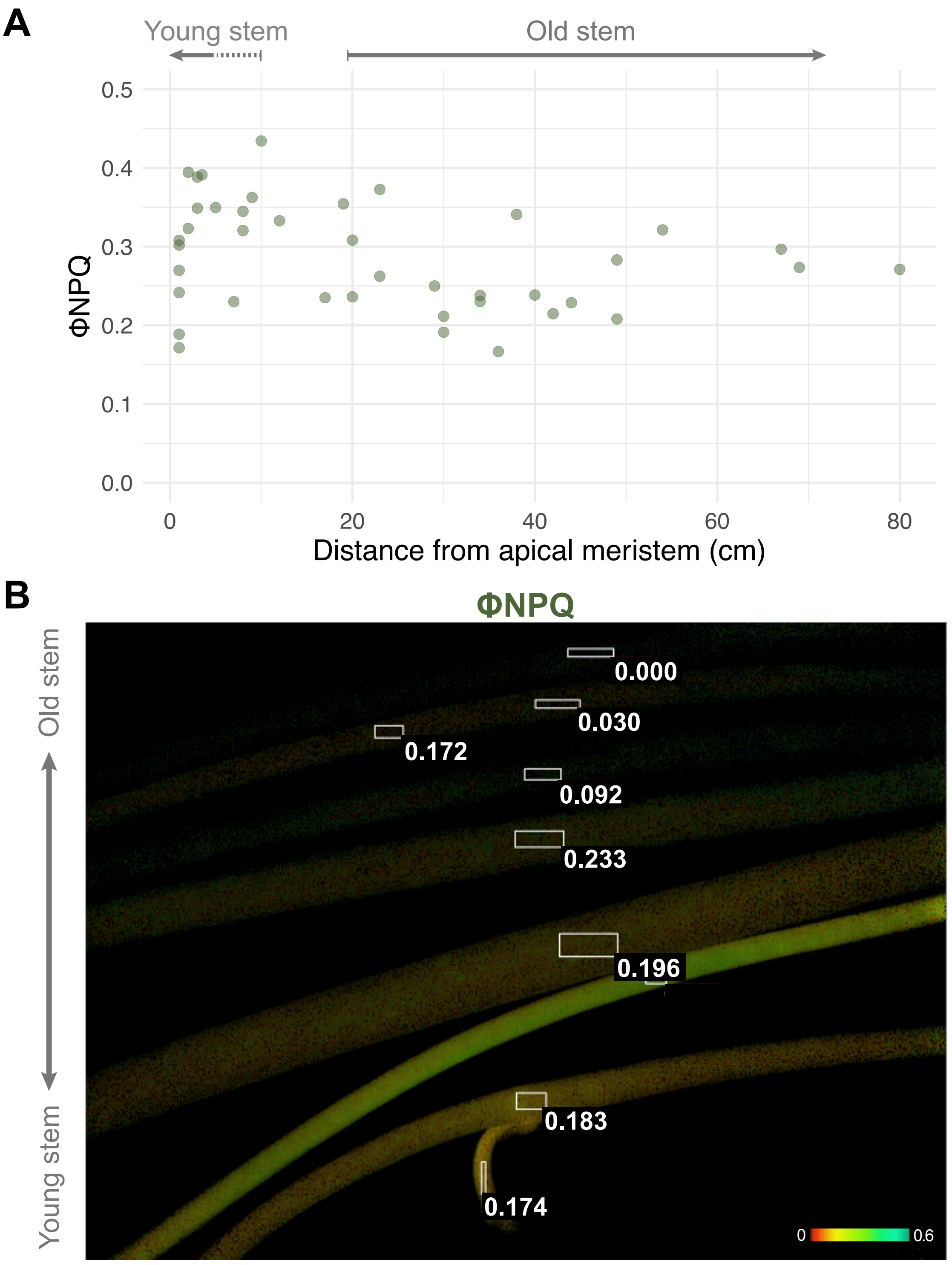

Appendix S1. Excel file with three sheets: (a) Pulse Amplitude Modulated chlorophyll fluorescence imaging (Imaging-PAM) raw data, (b) summary means by subgenus and ontogenetic stage; (c) summary means by species and ontogenetic stage.

Appendix S2. Excel file with three sheets: (a) raw pigment data, quantified by HPLC with external standards, (b) summary means by subgenus and ontogenetic stage; (c) summary means by species and ontogenetic stage.

Appendices S3–S8 are all tables of *P*-values. Cells are shaded red, yellow, light green, and dark green shading to indicate $P > 0.05$, $P < 0.05$, $P < 0.01$, and $P < 0.001$, respectively. Abbreviations: F_v/F_m = maximum quantum yield of open PSII centers; Φ_{PSII} = effective quantum yield of PSII; Φ_{NPQ} = proportion of xanthophyll-regulated non-photochemical quenching; l = *Ipomoea* leaf; sdlg = seedling; y = young stem; o = old stem; h = haustorium; f = flower; s = immature seed.

Appendix S3. *P*-value tables showing the results of pairwise Dunn tests between all ontogeny-subgenus pairwise combinations of F_v/F_m, Φ_{PSII}, and ΦNPQ, with a Benjamini and Hochberg correction for multiple tests. (Excel file with 3 sheets.)

Appendix S4. *P*-value tables showing the results of pairwise Dunn tests between all ontogeny-subgenus pairwise combinations of pigments, with a Benjamini and Hochberg correction for multiple comparisons. (Excel file with 12 sheets.)

Appendix S5. Tables of *P*-values generated by the post hoc Dunn tests comparing chlorophyll fluorescence parameters F_v/F_m, Φ_{PSII}, and ΦNPQ in different tissues from each clade, adjusted for multiple comparisons and summarized in Figure 2. (Excel file with 11 sheets.)

Appendix S6. Tables of *P*-values generated by the post hoc Dunn tests comparing pigment concentrations in different tissues from each clade, adjusted for multiple comparisons and summarized in Figures 4 and 5. (Excel file with 47 sheets.)

Appendix S7. Tables of *P*-values generated by the post hoc Dunn tests comparing chlorophyll fluorescence parameters F_v/F_m, Φ_{PSII}, and ΦNPQ in different tissues from each species of *Cuscuta* subgen. *Grammica*, adjusted for multiple comparisons and summarized in Figure S6 (Excel file with 25 sheets.)

Appendix S8. Tables of *P*-values generated by the post hoc Dunn tests comparing pigment concentrations in different tissues from each clade, adjusted for multiple comparisons and summarized in Figures S2 and S3. (Excel file with 61 sheets.)

References

Costea M, García MA, Stefanović S. 2015. A Phylogenetically based infrageneric classification of the parasitic plant genus *Cuscuta* (Dodders, Convolvulaceae). *Systematic Botany* **40**: 269–285.

García MA, Costea M, Kuzmina M, Stefanović S. 2014. Phylogeny, character evolution, and biogeography of *Cuscuta* (dodders; Convolvulaceae) inferred from coding plastid and nuclear sequences. *American Journal of Botany* **101**: 670–690.

Nakagawa S, Schielzeth H. 2012. A general and simple method for obtaining R^2 from generalized linear mixed-effects models. *Methods in Ecology and Evolution* **4**: 133–142.
